# Atlanto-axial morphometric measurements for age and sex estimation: a retrospective cone beam computed tomography study

**DOI:** 10.1007/s44445-026-00203-6

**Published:** 2026-07-01

**Authors:** Shubham M. Pawar, Junaid Ahmed, M. Archana, Nandita Shenoy, Nanditha Sujir, Komal Smriti, N. Srikant

**Affiliations:** 1 Department of Oral Medicine and Radiology, Manipal College of Dental Sciences Mangalore, Manipal Academy of Higher Education, Manipal, India; 2 Department of Oral Medicine and Radiology, Manipal College of Dental Sciences, Manipal Academy of Higher Education, Manipal, India; 3 Department of Oral Pathology and Microbiology, Manipal College of Dental Sciences Mangalore, Manipal Academy of Higher Education, Manipal, India

**Keywords:** Cone beam computed tomography, Sex estimation, Age, Forensic anthropology

## Abstract

Accurate determination of age and sex is fundamental in forensic anthropology, archaeology, and clinical diagnostics. Conventional methods are often limited by subjectivity and low precision. Cone Beam Computed Tomography (CBCT), with its high-resolution three-dimensional imaging, offers a reliable alternative for evaluating craniofacial structures. This study aimed to assess the utility of atlantoaxial morphometric parameters, particularly the atlantodental interval, in age and sex determination using CBCT. A retrospective observational study was conducted on 200 full-field CBCT scans (100 males, 100 females) of individuals aged 20–80 years. Scans meeting inclusion criteria were analyzed using standardized protocols. Eight parameters related to atlas (C1) and axis (C2) morphology and atlantodental intervals (AADI, PADI, LADI) were measured. Statistical analysis included ANOVA, independent t-tests, effect size estimation, and discriminant function analysis. Most parameters showed no significant variation across age groups, except posterior atlantodental interval (PADI), which demonstrated statistical significance. Sex-based comparisons revealed significantly greater atlas and axis dimensions in males (*p* < 0.01), indicating marked sexual dimorphism. Effect size analysis showed very large effects for atlas measurements and moderate to large effects for axis parameters. Discriminant analysis identified axis vertical length as the most reliable predictor of sex (accuracy: 82.5%), followed by axis anteroposterior length (72%). Atlantodental interval measurements showed limited discriminatory value. Atlantoaxial parameters exhibit selective age- and sex-related variations, with axis measurements demonstrating the highest utility for sex estimation. However, overall diagnostic accuracy remains moderate, indicating that these parameters should be used adjunctively. Further large-scale studies are required to validate their application in forensic and clinical settings.

## Introduction

A critical facet of human identification revolves around discerning an individual’s gender, serving as a fundamental factor in diverse identification methodologies (Capitaneanu et al. 2017). The combined determination of age and sex stands as a cornerstone in the development of a comprehensive biological identification profile for an individual (Capitaneanu et al. 2017).

Over the past few years, CBCT has garnered widespread recognition in dentistry due to its ability to produce three-dimensional (3D) data with reduced radiation exposure and cost, coupled with superior spatial resolution compared to conventional CT scanners (Miracle and Mukherji 2009). The high-resolution imaging capabilities and non-invasive nature of CBCT have prompted its widespread use in various medical disciplines, including forensic anthropology and clinical diagnostics (Issrani et al. 2022).

Understanding the age and sex of an individual from skeletal remains is crucial in forensic anthropology, archeology, and clinical diagnostics (Bašić et al. 2013). It forms the basis for constructing biological profiles, aiding criminal investigations, historical reconstructions, and medical diagnoses. However, traditional methods for age and sex determination often rely on subjective assessments and lack precision (Prokop-Piotrkowska et al. 2021). Therefore, there is a pressing need to explore novel approaches that offer more accurate and reliable results. This present study seeks to bridge the identified gap by examining the efficacy of craniofacial parameters, measured using CBCT, in ascertaining age and sex.

Craniofacial parameters such as the atlantodental interval have been identified as potential indicators of age and sex-related variations. However, their utility in forensic anthropology and clinical practice remains underexplored due to limited two-dimensional views that cover this region. By employing CBCT, which provides high-resolution 3D imaging of craniofacial structures, our study seeks to quantify these parameters accurately and investigate their correlations with age and sex.

The rationale underlying this study is multifaceted and encompasses several key considerations. Firstly, traditional methods for age and sex determination, such as visual inspection of skeletal remains or two-dimensional radiography, are often limited by subjectivity and lack of precision. CBCT offers advantages such as improved visualization, precise measurements, and non-invasiveness, making it an ideal tool for studying craniofacial parameters. By utilizing CBCT, this study aims to overcome the limitations of traditional methods and provide more reliable age and sex estimates (Magat and Ozcan 2019).

Secondly, the investigation of craniofacial parameters for age and sex determination has practical implications in forensic anthropology and archaeology (Nagare et al. 2018). Accurate estimation of age and sex from skeletal remains is essential for identifying individuals, especially in cases of mass disasters or historical investigations (Ubelaker and Khosrowshahi 2019).

Thirdly, in clinical practice, precise age and sex determination from craniofacial parameters can aid in diagnosing developmental anomalies, planning orthodontic treatments, and guiding surgical interventions (Dolci et al. 2023, Al-Taai et al. 2022).

While the atlantoaxial complex represents a structurally significant yet underutilized region, existing literature lacks a comprehensive evaluation of its morphometric parameters using CBCT. Given the limited evidence regarding the atlantoaxial region, this study seeks to address the question of whether the atlanto-axial morphometric measurements using CBCT can reliably aid in age and sex estimation.

This study aimed to assess the atlanto axial morphometric parameters in age and sex estimation using CBCT.

## Material and methods

Individuals who reported to the Department of Oral Medicine and Radiology, Manipal College of Dental Sciences Mangalore for full FOV CBCT scans between September 2019 and February 2024, fulfilling the study requirement, were included in this observational retrospective study. This study was approved by the Institutional Ethics Committee, Manipal College of Dental Sciences Mangalore (Protocol Ref No: 22034).

The sample size calculation was based on the study conducted by Osmotherly PG et al. (2013), which investigated the relationship between atlanto-dental interval measurements and age and sex in an adult cohort.

Assuming the alpha error (α) to be 1%, power (1 – β) to be 80% and clinically significant difference = 2 units; the calculated sample size was 96 subjects per group (males and females). To compensate for approximately 4% measurement error, an additional 4 samples per group were included.

Therefore, the final sample size was rounded to 100 subjects for each group, resulting in a total sample size of 200 CBCT scans.

### Inclusion criteria

1. Patients above the age of 20 years.
2. Full FOV CBCT scans.
3. Scans exhibiting high resolution and free from any artifacts.

### Exclusion criteria

1. Patients who are below 20 years of age.
2. Patients with skull bone abnormalities (congenital or acquired), degenerative changes or abnormalities in the atlas – axis region.
3. Patients who have a history of trauma or fractures of the skull.

All scans were obtained using a Planmeca ProMax 3D Mid CBCT unit (Planmeca, Helsinki, Finland) at 90 kV and 8 mA, with a 200 × 170 mm field of view and 0.4 mm voxel resolution. The acquired images were evaluated using Romexis software (version 7).

Full FOV CBCT scans acquired as a part of the routine dental investigations for orthodontic evaluation, and routine scans that fit into the inclusion and exclusion criteria were assessed. To avoid bias, the radiographs were coded, and the observer performing the measurements was blinded to the age and sex of the participants. The radiographs were assessed by two independent observers. Inter-rater reliability was estimated by using the Intraclass Correlation Coefficient (ICC) two-way mixed effects model.

Head orientation was standardized prior to measurement by aligning the CBCT volume in multiplanar reconstruction such that the Frankfort horizontal plane was parallel to the floor, the midsagittal plane was centered and perpendicular. All measurements were performed using multiplanar reconstruction views (axial, sagittal, and coronal) with synchronized cursor positioning to ensure spatial accuracy. Measurement planes were defined by orienting the sagittal slice through the midline passing through the odontoid process, while axial and coronal planes were adjusted perpendicular to this reference. Anatomical landmarks, including the anterior and posterior arches of the atlas (C1), the dens of the axis (C2), and the lateral masses, were identified based on consistent radiographic criteria and verified across all three planes to minimize observer variability.

**Fig. 1:**
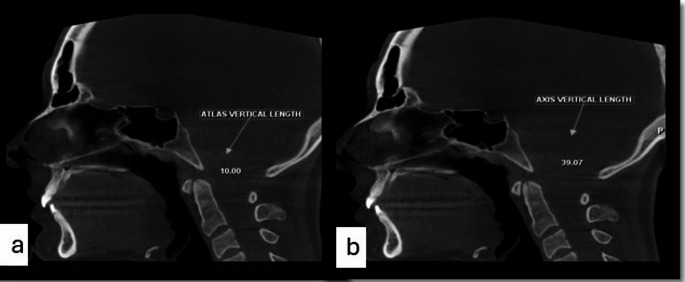
Sagittal view showing measurement of the vertical length of the atlas (C1) and axis (C2)

**Fig. 2:**
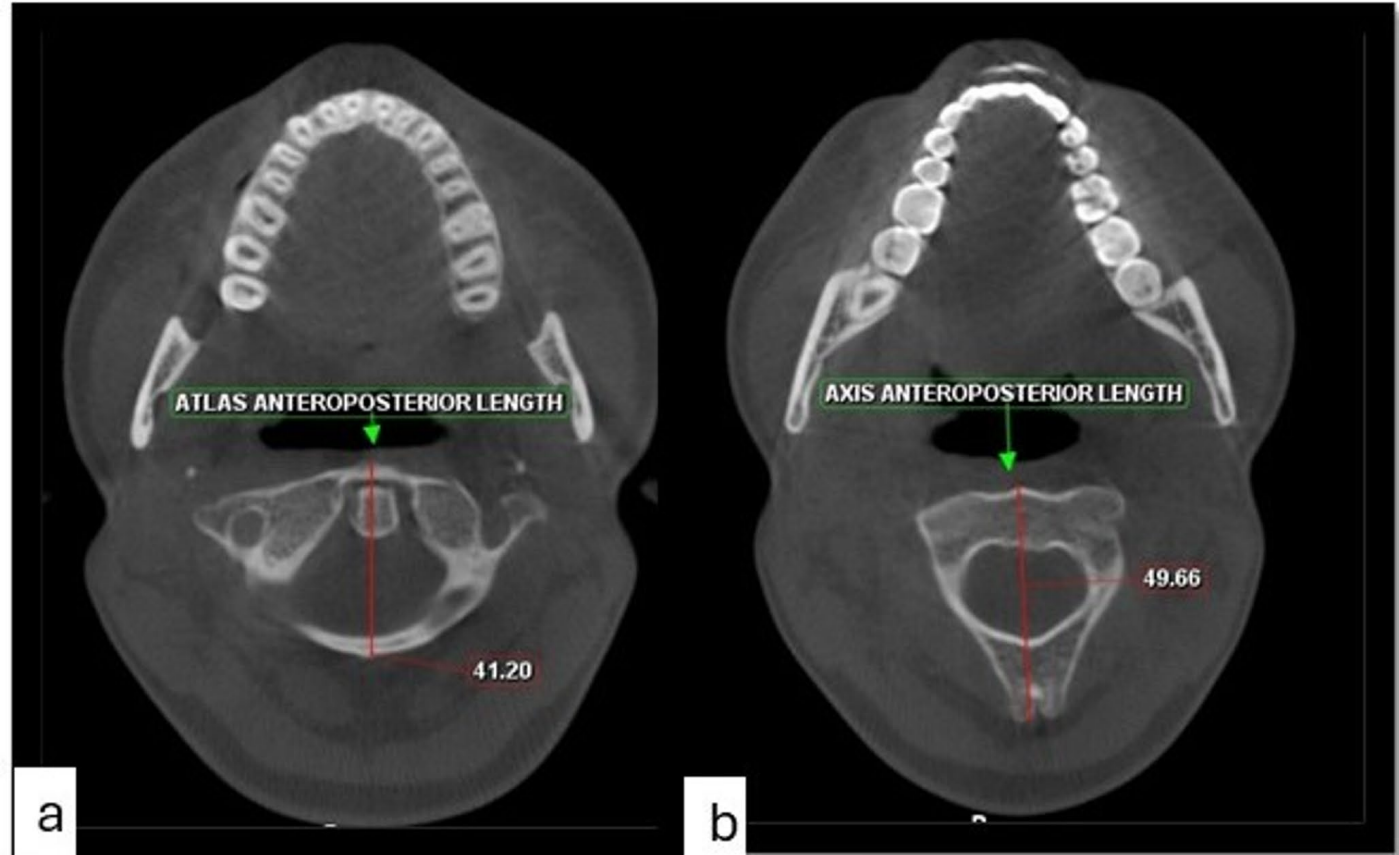
Axial view showing measurement of atlas anteroposterior length, which is from the anterior edge of the anterior arch to the posterior edge of the posterior arch of the atlas (C1) and axis (C2)

**Fig. 3:**
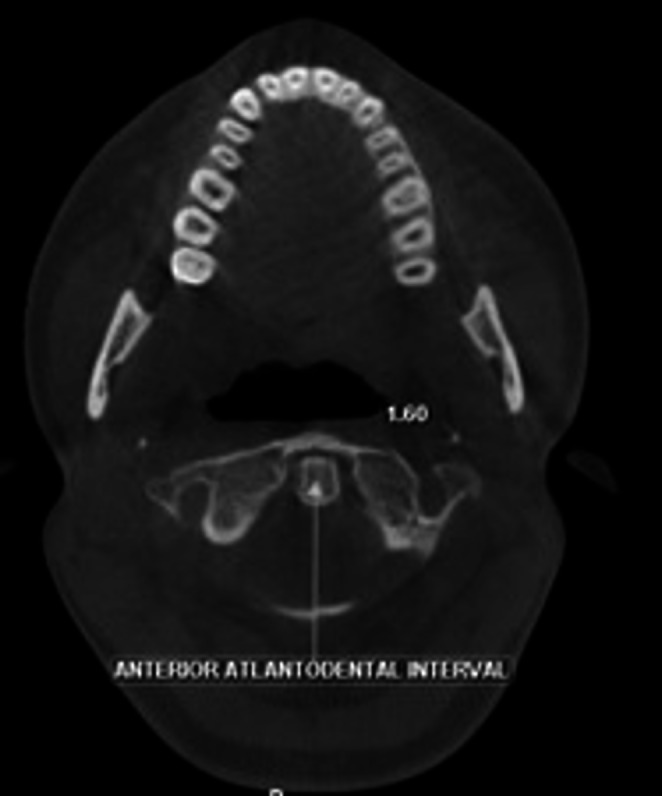
Axial view showing measurement for Anterior atlantodental interval, which is the distance between the anterior edge of the dens and the posterior edge of the anterior arch of the atlas

**Fig. 4:**
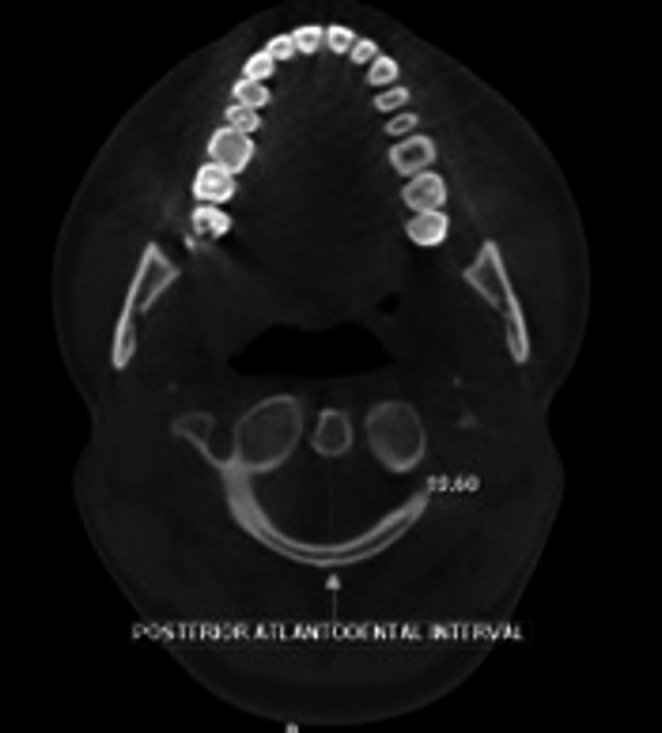
Axial view showing measurement for Posterior atlantodental interval, which is the distance between the posterior edge of the dens and the anterior edge of the posterior arch of the atlas

**Fig. 5:**
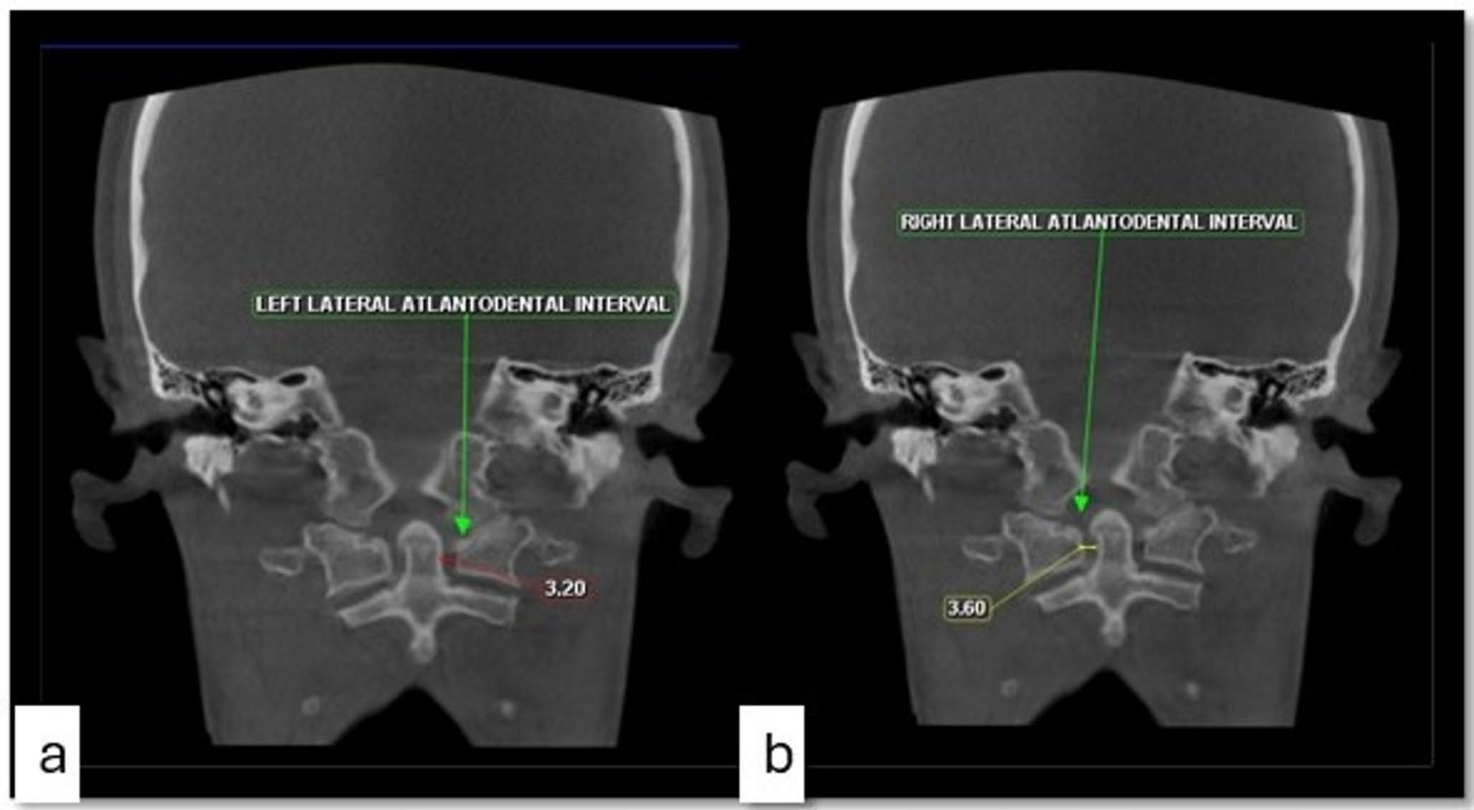
Coronal view showing measurement of Left and right lateral atlanto-dental interval, which is calculated by drawing a line between the lateral surface of the dens and the medial surface of the lateral mass of C1

To analyse the Atlantodental region, eight parameters were assessed using CBCT. Atlas (C1) and axis (C2) morphology were assessed using C1 vertical length (C1 Ver) (Fig. 1), C1 antero-posterior length (C1 AP) (Fig. 2), C2 vertical length (C2 Ver) (Fig. 1), C2 antero-posterior length (C2 AP) (Fig. 2). The Atlantodental interval(ADI) was assessed by measuring the Anterior Atlantodental interval (AADI) which is defined as “the distance between the anterior edge of dens and the posterior edge of the anterior arch of the atlas”(Fig. 3), Posterior atlantodental interval (PADI) defined as “distance between the posterior edge of the dens and the anterior edge of the posterior arch of the atlas”(Fig. 4), and Lateral atlantodental interval (LADI) was measured in coronal plane at C1 mid-lateral mass level which is calculated by drawing line between medial surface of lateral mass of C1 and lateral surface of dens (Fig. 5) as described by *Cesur E*.

## Results

SPSS version 20.0 was used to perform statistical analysis. Differences in recorded variables across age groups (20–35, 36–50, 51–65, and 66–80 years) were assessed via one-way ANOVA with F-tests and Welch’s ANOVA (W-test) where applicable. Post hoc tests identified pairwise mean differences between groups. Independent t-tests were used to assess sex-based differences among variables, and stepwise discriminant function analysis was employed to classify sex. Statistical significance was set at *p* < 0.05.

A total of 200 scans consisting of 8 parameters were radiographically evaluated in the present study.

All the scans were of patients between the ages of 20 and 80 years. The sample was grouped into 4, i.e., (20–35), (36–50), (51–65), and (66–80) years.

Selected scans were assessed for the Atlas vertical height, Atlas anteroposterior length, axis vertical dimension, axis anteroposterior length, Anterior and Posterior Atlantodental Interval.

### Comparison of parameters across age groups and post hoc analysis

Comparison of morphometric parameters across age groups (20–35, 36–50, 51–65, and 66–80 years) revealed variable trends (Table 1).

**Table 1:** Mean of study variables and test for significant difference of various parameters between the different age groups

	20–35 (*n* = 50)	36–50 (*n* = 50)	51–65 (*n* = 50)	66–80 (*n* = 50)	Effect Size	Welch Statistics	*P* value
Atlas vertical length	10.17 ± 0.43	10.11 ± 0.4	10.19 ± 0.41	10.14 ± 0.46	0.006	0.439	0.725
Atlas AP length	39.87 ± 0.8	40.35 ± 0.96	39.65 ± 0.81	40.07 ± 1.28	0.067	5.516	**0.001**
Axis vertical	40.11 ± 1.7	40.06 ± 0.85	40.35 ± 1.63	40.39 ± 0.78	0.013	1.569	0.201
Axis Ap length	45.46 ± 1.05	45.57 ± 1.07	45.66 ± 1.4	45.52 ± 1.07	0.004	0.241	0.868
AADI	1.62 ± 0.11	1.63 ± 0.1	1.61 ± 0.15	1.61 ± 0.11	0.005	0.385	0.764
PADI	19.53 ± 0.15	19.6 ± 0.16	19.42 ± 0.44	19.51 ± 0.3	0.048	3.739	**0.013**
R LADI	3.7 ± 0.16	3.65 ± 0.16	3.76 ± 0.2	3.67 ± 0.11	0.064	3.49	**0.018**
L LADI	3.62 ± 0.18	3.58 ± 0.17	3.65 ± 0.14	3.63 ± 0.15	0.020	1.322	0.271

Comparison of atlanto-axial morphometric parameters among the four age groups (20–35, 36–50, 51–65, and 66–80 years) revealed statistically significant differences for Atlas AP length, PADI, and right LADI. Atlas AP length differed significantly across age groups (Welch statistic = 5.516, *p* = 0.001), with the highest mean value observed in the 36–50 years group (40.35 ± 0.96 mm) and the lowest in the 51–65 years group (39.65 ± 0.81 mm). Similarly, PADI demonstrated significant age-related variation (Welch statistic = 3.739, *p* = 0.013), with slightly higher values in the 36–50 years group (19.60 ± 0.16 mm) and lower values in the 51–65 years group (19.42 ± 0.44 mm). Right LADI also showed significant differences among age groups (Welch statistic = 3.49, *p* = 0.018), with the highest mean recorded in the 51–65 years group (3.76 ± 0.20 mm) and the lowest in the 36–50 years group (3.65 ± 0.16 mm).

No statistically significant differences were observed for Atlas vertical length (*p* = 0.725), Axis vertical length (*p* = 0.201), Axis AP length (*p* = 0.868), AADI (*p* = 0.764), or left LADI (*p* = 0.271), indicating relative stability of these measurements across age categories. As most parameters did not reach statistical significance, post hoc analysis was not performed except where indicated.

### Comparison between male and female groups

Table 2 presents the comparison of atlanto-axial morphometric measurements between male and female groups. Among the evaluated parameters, Axis vertical length and Axis anteroposterior (AP) length demonstrated statistically significant sex differences. Males exhibited significantly greater Axis vertical length (40.91 ± 0.68 mm) than females (39.56 ± 1.43 mm) (t = 8.504, *p* < 0.001), with a large effect size (Cohen’s d = 1.12). Similarly, males showed significantly greater Axis AP length (46.07 ± 1.03 mm) compared to females (45.05 ± 1.03 mm) (t = 7.013, *p* < 0.001), also with a large effect size (Cohen’s d = 1.03).

**Table 2:** Comparison of variables of the parameters based on sex using an independent t-test

	Male (*n* = 99)	Female (*n* = 101)	t	*P*	Cohens D
Atlas vertical length	10.17 ± 0.41	10.14 ± 0.44	0.533	0.595	0.425397
Atlas AP length	40.09 ± 0.93	39.88 ± 1.07	1.489	0.138	1.004717
Axis vertical	40.91 ± 0.68	39.56 ± 1.43	8.504	< 0.001	1.123583
Axis AP length	46.07 ± 1.03	45.05 ± 1.03	7.013	< 0.001	1.02999
AADI	1.63 ± 0.12	1.6 ± 0.11	1.817	0.071	0.11721
PADI	19.5 ± 0.35	19.54 ± 0.23	-0.954	0.341	0.2942
R LADI	3.7 ± 0.16	3.7 ± 0.17	-0.097	0.923	0.165198
L LADI	3.61 ± 0.16	3.63 ± 0.16	-0.757	0.45	0.160846

No statistically significant differences were observed between males and females for Atlas vertical length (*p* = 0.595), Atlas AP length (*p* = 0.138), AADI (*p* = 0.071), PADI (*p* = 0.341), right LADI (*p* = 0.923), or left LADI (*p* = 0.450). Although Atlas AP length showed a relatively large Cohen’s d value, the difference did not reach statistical significance. The remaining parameters demonstrated small effect sizes, indicating minimal sex-related variation.

Overall, the findings suggest that Axis dimensions exhibit marked sexual dimorphism, with males having significantly larger measurements than females, whereas most atlas and atlanto-dental interval parameters remain comparable between the sexes. These results indicate that Axis morphometric measurements may have greater utility in sex estimation than atlas-related dimension.

### Effect size interpretation for age categories

The effect sizes (η²) observed across the age categories ranged from negligible to moderate. The largest effect sizes were noted for Atlas AP length (η² = 0.067) and right LADI (η² = 0.064), indicating a moderate influence of age on these parameters. PADI (η² = 0.048) demonstrated a small-to-moderate effect size, suggesting some age-related variation. In contrast, Atlas vertical length (η² = 0.006), Axis vertical length (η² = 0.013), Axis AP length (η² = 0.004), AADI (η² = 0.005), and left LADI (η² = 0.020) showed small or negligible effect sizes, indicating minimal practical differences across age groups. Overall, age appeared to have the greatest influence on Atlas AP length, PADI, and right LADI, while its effect on the remaining morphometric parameters was limited.

### Effect size interpretation for sex (independent samples t-test, cohen’s d)

Cohen’s d analysis showed the largest effect sizes for Axis vertical length (d = 1.12), Axis AP length (d = 1.03), and Atlas AP length (d = 1.00), indicating substantial sex-related differences. Atlas vertical length (d = 0.43) demonstrated a small-to-moderate effect, while PADI (d = 0.29) showed a small effect. AADI (d = 0.12), right LADI (d = 0.17), and left LADI (d = 0.16) exhibited negligible effect sizes. Overall, Axis measurements showed the strongest sexual dimorphism and may have greater value for sex estimation, whereas atlanto-dental and lateral atlanto-dental interval measurements demonstrated limited sex-related variation.

### Discriminant function analysis for sex discrimination

Stepwise discriminant function analysis identified the parameter(s) with the highest accuracy as follows: axis vertical length (82.50%), followed by axis Antero-posterior length (72%), left Lateral Atlantodental Interval (54.50%), Anterior Atlantodental Interval (53.50%), Posterior Atlantodental Interval (52.50%), atlas Antero-posterior length (51.50%), atlas vertical length (51%), and right Lateral Atlantodental Interval (51%). (Table 3)

**Table 3:** Discriminant function analysis for discriminating sex

Parameter	Equation	Percentage of Females correctly classified	Percentage of Males correctly classified	overall accuracy	Male Centroid	Female Centroid	Sectioning point	Demarcating point
Atlas vertical length	Discriminant function (D)=-23.866+(2.351) x (Atlas vertical length)	63.6	38.6	51.00%	0.038	-0.037	0.000125	10.15142
Atlas Anteroposterior length	Discriminant function (D)=-39.798+(0.995) x (Atlas AP length)	48.5	54.5	51.50%	0.106	-0.104	-5E-05	39.99799
Axis vertical	Discriminant function (D)=-35.803+(0.89) x (Axis vertical)	81.8	83.2	82.50%	0.607	-0.595	-1E-05	40.22809
Axis Anteroposterior length	Discriminant function (D)=-44.226+(0.971) x (Axis Anteroposterior length)	72.7	71.3	72.00%	0.501	-0.491	4E-05	45.54686
AADI	Discriminant function (D)=-13.789+(8.532) x(AADI)	59.6	47.5	53.50%	0.13	-0.127	0.000215	1.616151
PADI	Discriminant function (D)=-66.336+(3.399) x(PADI)	44.4	60.4	52.50%	-0.068	0.067	0.000175	19.51633
Right LADI	Discriminant function (D)=-22.378+(6.053) x (R LADI)	55.6	46.5	51.00%	-0.007	0.007	7E-05	3.69701
Left LADI	Discriminant function (D)=-22.503+(6.217) x (L LADI)	54.5	54.5	54.50%	-0.054	0.053	3.5E-05	3.619591

### Interobserver reliability

Inter-rater reliability was estimated by using the Intraclass Correlation Coefficient (ICC) two-way mixed effects model. All measurements resulted in good to excellent agreement among the observers. The ICC coefficients that ranged from 0.672 to 0.996 indicated acceptable reproducibility. Among the parameters evaluated, Axis vertical proved to be the most reliable variable, over the other studied parameters, with an ICC of 0.996 (95% CI: 0.983–0.999; *p* < 0.001). Axis AP length was rated second in reliability with an ICC of 0.968 (95% CI: 0.877–0.992; *p* < 0.001). Other parameters analyzed for reliability were Atlas vertical length (ICC = 0.949, 95% CI: 0.811–0.987; *p* < 0.001) and Atlas AP length (ICC = 0.932, 95% CI: 0.753–0.983; *p* < 0.001). AADI also revealed good agreement (ICC: 0.846; 95% CI: 0.496–0.959; *p* < 0.001). The PADI (ICC = 0.783, 95% CI: 0.343–0.941; *p* = 0.002), and L LADI (ICC = 0.706, 95% CI: 0.180–0.918; *p* = 0.008) measurement methods show moderate to good agreement. The lowest agreement was found for R LADI (ICC = 0.672 (95% CI: 0.117–0.907; *p* = 0.012). There was a statistically significant inter-observer agreement for all parameters (*p* < 0.05).

## Discussion

The present study evaluated 200 CBCT scans to assess age and sex-related variations in atlantoaxial morphometric parameters. Among the age groups, statistically significant differences were observed in atlas anteroposterior length, posterior atlantodental interval (PADI), and right lateral atlantodental interval (R LADI), while other parameters did not show significant variation.

Sex-based comparison revealed that axis vertical and axis anteroposterior lengths were significantly greater in males, indicating notable sexual dimorphism in axis measurements. In contrast, atlas dimensions and atlantodental intervals did not demonstrate significant sex differences.

Discriminant function analysis identified axis vertical length as the most reliable parameter for sex determination, with the highest accuracy (82.5%), followed by axis anteroposterior length (72%). The remaining parameters showed relatively low discriminatory power, with accuracies close to chance level. Overall, the findings suggest that while certain atlantoaxial parameters exhibit age- and sex-related variations, their utility in isolation for reliable identification is limited.

A high level of inter-observer reliability was observed for all measurements (ICC = 0.672–0.996; *p* < 0.05), with Axis vertical length demonstrating the highest reproducibility (ICC = 0.996), indicating that the evaluated CBCT-based atlantoaxial morphometric parameters are reliable and reproducible for forensic assessment.

The complexity of the bone structure in the head and neck region, along with the unique characteristics they possess at various life stages due to embryological development, underscores their significance in surgical procedures and techniques related to human identification (Mello-Gentil and Souza-Mello 2021).

Sex determination with osteological and dental analyses has traditionally involved collaboration among dentistry, forensic medicine, and anthropology due to their overlapping interests in these domains. The methods for estimating sex based on cranial and dental characteristics are highly specific to populations, underscoring the importance of their application and validation across diverse populations (Beschiu et al. 2022).

The main objective of our study was to explore the usefulness of atlantoaxial morphometric parameters derived from CBCT for determining both age and sex. The parameters under investigation were atlas and axis vertical length, anteroposterior lengths of atlas and axis, and atlanto-dental interval measurements. Craniofacial parameters derived from imaging modalities like CBCT hold promise for age and sex determination in forensic and medical settings. The findings in the present study have shown sexual dimorphism in atlantoaxial features.

In the cervical vertebrae, the atypical atlas (C1) and axis (C2) prove particularly valuable for sex estimation. The atlas articulates directly with the skull base, where males typically endure greater biomechanical stress than females owing to their higher muscle mass, bone density, and brain weight. As a result, features such as the upper and lower articular surfaces and the vertebral foramen area tend to appear more pronounced in males (Mello-Gentil and Souza-Mello 2021).

The present findings are in partial agreement with the study by Magat and Ozcan (2019), who reported that odontoid process morphometry exhibits significant sexual dimorphism and can be useful for forensic identification (Magat and Ozcan 2019). Similarly, the current study found that Axis vertical length and Axis AP length were significantly greater in males than females, with large effect sizes and high discriminant accuracy, supporting their utility in sex estimation. However, unlike their findings suggesting an association with age estimation, the present study observed no significant age-related differences in axis dimensions, indicating that these measurements remain relatively stable across adult age groups. This discrepancy may be attributed to differences in study design, population characteristics, and the specific morphometric parameters evaluated. Both studies support the forensic value of axis-related measurements for sex determination, while their role in age estimation appears limited in adult populations.

The Axis vertical length and Axis anteroposterior (AP) length were found to be notably greater and significant in males compared to females. Zanutto et al. in 2021 reported that male CBCT scans of axis sagittal length, width, and transverse diameter are significantly higher compared to those of females (Zanutto et al. 2021). Additionally, the Atlas anteroposterior (AP) length demonstrated significant differences in the 36–55 age group compared to other age groups in our study but did not show a significant relationship between males and females.

Cesur et al. reported strong associations between facial dimensions across sagittal, vertical, and transverse planes, as well as facial position, and the morphology of the atlas and axis. These findings indicate that cervical vertebral growth is governed by both inherent developmental mechanisms and extrinsic influences such as functional adaptation, head posture, and craniofacial configuration (Cesur et al. 2020). 

The development, fusion trajectory, and pubertal growth of the axis dens established it as a key anatomical contributor to sexual dimorphism in adulthood. A study conducted with the Japanese population analysed CT images of the axis, resulting in the creation of a discriminant function. This function attained an impressive accuracy of 92.9% in estimating sex based on the characteristics observed in the axis (Goel 2021). Using discriminant function analysis in our study, the Axis vertical length correctly classified 81.8% of females and 83.2% of males, while the Axis Anteroposterior length classified females and males with an accuracy of 72.7% and 71.3%, respectively.

The atlantoaxial joint is commonly afflicted by trauma, arthropathies, and neoplasms, prompting numerous studies to diagnose instability in this region (Yoon et al. 2015). By acknowledging potential pathologies affecting the atlantoaxial joint, researchers can better interpret any observed variations in the craniofacial parameters being studied, potentially aiding in more accurate age and sex determinations. The joint offers rotation, flexion, and extension movements. The sole validated parameter for confirming the existence of atlantoaxial instability is the pathological change observed in the atlantodental interval on dynamic flexion-extension images of the craniovertebral junction (Yang et al. 2014).

With aging, there is a gradual reduction in articular cartilage volume between the odontoid process and the anterior arch of the atlas. Osmotherly et al. in 2013 found no sex-related association with ADI across various age groups, consistent with our findings. They suggested that ADI values might decrease with age, particularly in cases of minor clinical instability, highlighting the significance of considering age in such evaluations (Osmotherly et al. 2013).

Wu et al. in 2019 reported no statistically significant differences among age and sex groups in cross-sectional CT scans of the anterior atlantodental interval (AADI) (Wu et al. 2019). Likewise, we did not observe any significant differences in AADI among age and sex groups in our analysis.

Yoon et al. reported average Posterior atlantodental interval (PADI) values using multidetector computed tomography (MDCT) as 18.0 ± 2.1 (13.0–23.7) mm (Yoon et al. 2015). In our study, the average PADI values were 19.5 ± 0.35 for males and 19.54 ± 0.23 for females. Our study identified statistically significant differences in PADI among the 36–50 and 51–65 age groups. According to Yoon et al., PADI has been linked to the presence and severity of paralysis and is a crucial predictor of neurological recovery post-surgery in rheumatoid arthritis patients. PADI narrowing may impair blood flow in the anterior spinal, vertebral, and basilar arteries, independent of spinal cord compression (Yoon et al. 2015). As most vertebral growth is completed by early adulthood, many atlantoaxial dimensions demonstrate minimal changes with advancing age, resulting in the relatively stable measurements observed in the present study. This pattern suggests that structural dimensions of the axis are influenced predominantly by sex, whereas age-related effects are generally subtle and limited to specific atlantoaxial measurements (Yoon et al. 2015). 

In 2018, Mendenhall reported insignificant differences in the lateral atlantodental interval (LADI) values measured using CT among age groups and between males and females. Interestingly, LADI asymmetry emerged as a valuable and consistent CT measurement, remaining stable throughout childhood development irrespective of gender (Mendenhall et al. 2018). Similarly, our study did not find any significant gender disparities in the LADI values. Nevertheless, we observed significant differences in right LADI values among different age groups. Specifically, the right LADI values differed significantly between the age groups of 36–50 and 51–65, as well as between 51 and 65 and 66–80.

The findings of this study hold promise for enhancing radiological protocols aimed at determining age and sex, offering clinicians a more precise and thorough visualization of craniofacial structures. By elucidating age-related alterations in craniofacial parameters, this research may assist orthodontic interventions, leading to more precise alignment corrections and functional enhancements. Moreover, by refining methods for evaluating craniofacial parameters through CBCT, this study has the potential to bolster the accuracy of forensic investigations, aiding in the resolution of legal and humanitarian cases. The clinical significance of this research lies in its ability to advance diagnostic accuracy, refine treatment planning, and improve patient outcomes.

Given that only a limited number of parameters showed statistically significant associations and that the overall classification accuracy is moderate, this approach may be best applied as a supplementary tool alongside established forensic age and sex estimation methods. These measurements may serve as adjunctive rather than standalone indicators for age and sex estimation.

A major limitation of our study is the absence of true random sampling. Obtaining true random sampling, wherein every population member has an equal probability of selection, presents significant challenges. Instead, we employed nonprobability sampling methods, specifically quota sampling. Hence, the outcomes of our study cannot be extrapolated to the broader population. Additionally, as our sample consisted solely of individuals from Mangalore or the Dakshina Kannada district, the generalizability of our findings to other geographic regions may be limited. Another limitation is that age-related trends are inferred from comparisons between predefined age groups instead of evaluating actual aging trajectories over time. The manuscript currently relies solely on internal analysis and the absence of external validation limits the robustness and generalizability of the predictive model.

Numerous studies have reported variations in findings regarding the relationship between craniofacial parameters, sex, and age. Factors such as population demographics, imaging modalities, and methodological differences contribute to these variations. Hence, further research and validation are necessary to establish robust guidelines and address variability in findings. Further studies can enhance the reliability and applicability of craniofacial parameter analysis, contributing to improved patient outcomes and more effective forensic investigations.

## Conclusions

The present study demonstrated that only a limited number of atlantoaxial parameters exhibited statistically significant associations with age and sex, indicating a modest potential for their use in age and sex estimation. However, the overall discriminant accuracy observed was moderate, suggesting that these parameters alone may not be sufficient for reliable identification.

Therefore, the findings of this study should be interpreted as exploratory. Further research involving larger, more diverse, and well-balanced populations is necessary to validate these observations and improve their applicability. Establishing more robust and standardized guidelines through such studies will be essential to enhance the reliability of atlantoaxial measurements in forensic and clinical settings.

## Data Availability

The data that support the findings of this study are available from the corresponding author upon reasonable request.
